# Preservation affinity in consensus modules among stages of HIV-1 progression

**DOI:** 10.1186/s12859-017-1590-3

**Published:** 2017-03-20

**Authors:** Sk Md Mosaddek Hossain, Sumanta Ray, Anirban Mukhopadhyay

**Affiliations:** 1 Department of Computer Science and Engineering, Aliah University, Kolkata, West Bengal, 700156 India; 2 Department of Computer Science and Engineering, University of Kalyani, Kalyani, West Bengal, 741235 India

**Keywords:** Gene co-expression network, Module eigengene, Hierarchical clustering, Consensus modules, Immune regulatory genes

## Abstract

**Background:**

Analysis of gene expression data provides valuable insights into disease mechanism. Investigating relationship among co-expression modules of different stages is a meaningful tool to understand the way in which a disease progresses. Identifying topological preservation of modular structure also contributes to that understanding.

**Methods:**

HIV-1 disease provides a well-documented progression pattern through three stages of infection: acute, chronic and non-progressor. In this article, we have developed a novel framework to describe the relationship among the consensus (or shared) co-expression modules for each pair of HIV-1 infection stages. The consensus modules are identified to assess the preservation of network properties. We have investigated the preservation patterns of co-expression networks during HIV-1 disease progression through an eigengene-based approach.

**Results:**

We discovered that the expression patterns of consensus modules have a strong preservation during the transitions of three infection stages. In particular, it is noticed that between acute and non-progressor stages the preservation is slightly more than the other pair of stages. Moreover, we have constructed eigengene networks for the identified consensus modules and observed the preservation structure among them. Some consensus modules are marked as preserved in two pairs of stages and are analyzed further to form a higher order meta-network consisting of a group of preserved modules. Additionally, we observed that module membership (MM) values of genes within a module are consistent with the preservation characteristics. The MM values of genes within a pair of preserved modules show strong correlation patterns across two infection stages.

**Conclusions:**

We have performed an extensive analysis to discover preservation pattern of co-expression network constructed from microarray gene expression data of three different HIV-1 progression stages. The preservation pattern is investigated through identification of consensus modules in each pair of infection stages. It is observed that the preservation of the expression pattern of consensus modules remains more prominent during the transition of infection from acute stage to non-progressor stage. Additionally, we observed that the module membership values of genes are coherent with preserved modules across the HIV-1 progression stages.

**Electronic supplementary material:**

The online version of this article (doi:10.1186/s12859-017-1590-3) contains supplementary material, which is available to authorized users.

## Background

Acquired Immunodeficiency Syndrome (AIDS) is one of the cataclysmic diseases that have impaired the human species for decades. In spite of the enormous amount of efforts and resources employed to its study and even after thirty-three years of unveiling of the fact that Human Immunodeficiency Virus (HIV) as the cause of AIDS, there is still no effective vaccine and no cure for this disease [1–3].

After initial infection, a person may not experience any symptom or may undergo a brief period of influenza-like illness, including fever, headache, rash or a sore throat. Typically, this is collocated with a prolonged period of time with no symptoms. As the infection develops, it interacts more with the immune system, intensifying the danger of common infections like tuberculosis, as well as other expedient infections, and tumors that seldom endanger people who have functioning immune systems (http://www.who.int/mediacentre/factsheets/fs360/en/). These late, defenseless to grievous infections are categorized as AIDS. People often observed substantial weight loss at this stage (http://www.cdc.gov/hiv/basics/whatishiv.html).

There are three main stages of HIV infection: the acute stage (also known as primary HIV infection or acute retroviral syndrome), chronic stage (this stage is sometimes called “asymptomatic HIV infection”, “chronic infection” or “clinical latency stage”), and AIDS [4]. In the acute stage, the initial period following the contraction of HIV, it takes 2-3 weeks after infection until the copy number of HIV-1 virus increases, and the number of CD4+ T (T helper) cells remarkably reduces [5]. However, usually, patients infected with HIV-1 overcome from the acute stage without any treatments within 3-6 weeks and have a clinical latency period of 8 to 10 years (chronic stage) [6].

Although mostly there are few or no symptoms at first and CD4+ T cell count is almost recovered during the clinical latency stage, it has been discovered that immune damage occurs persistently [7]. A small proportion (about 5 to 8%) of HIV-infected patients maintain high levels of CD4+ T cells (T helper cells) without antiretroviral therapy and stay clinically stable for decades. They are called HIV controllers or long-term non-progressors (LTNP) [8]. Nevertheless, the most of the HIV-1 infected patients have a perceptible viral load and in lack of treatment will eventually advance to AIDS, a stage where the CD4+ T cell count falls below 200 cells / *μ*L, and hence T cell-mediated immunity fails to defend the body from pathogens [9].

In recent years, researchers are practicing an extensive use of DNA microarray technology to analyze the expression levels of thousands of genes simultaneously to understand the rationales of cellular systems, molecular networks, disease mechanisms, etc. To reveal system-level properties of genes, construction and analysis of biological networks have been extensively used in [10–13]. Examples of such biological networks are gene regulatory networks, protein-protein interaction networks, metabolic networks, signaling networks, gene co-expression networks, etc. Amidst these biological networks, gene co-expression networks have numerous advantages [14], and empower us to endure a global overview of different diseases. In a co-expression network, genes are interconnected to each other on the basis of the resemblance of their expression profiles and such co-expressed genes tend to participate in the same pathway or form complexes [15, 16] that perform specific functions.

For analyzing the similarities and heterogeneity in network structures through co-expression modules, quite a significant number of computational methodologies have been put forward in [17–19]. To discover the preservation patterns in modules between the human brain and blood tissue, Cai et al., introduced a novel framework in [20]. Conservation and evolution of gene co-expression networks across the human and chimpanzee brains have been studied by Oldham et al., in [17]. To reveal the association within the co-expression modules through eigengene networks, a revolutionary framework has been introduced by Langfelder et al. [21]. Ray et al. [22] proposed a novel framework to discover topological pattern changes in gene co-expression modules through eigengene networks among different stages of HIV-1 progression using a rank aggregation scheme. A novel framework has been proposed in [23] for discovering the preservation and expression pattern changes in co-expressed modules across three stages of HIV-1 disease progression through an eigengene-based analysis.

In this article, we have developed a novel framework to study the preservation and changes of modular structure in the gene co-expression networks across three stages of HIV-1 disease progression through eigengene-based approach. Initially, we have compiled three separate co-expression networks through Weighted Gene Co-Expression Network Analysis (WGCNA) framework [24] for three stages of HIV-1 disease progression. Next, consensus modules are identified by considering each pair of stages at a time. We have also searched for the immune regulatory genes which are preserved and not preserved among the HIV-1 infection stages across the consensus modules. Additionally, to investigate the topological characteristics of all the shared genes belonging to those consensus modules, we have computed their degree and betweenness centrality and identified the most significant Gene Ontology (GO) Terms and KEGG Pathways associated with them. The overlaps between each pair of consensus modules are investigated through an overlap score. The preservation patterns of the identified consensus modules are then discovered by using an eigengene-based measures. For consensus modules between a pair of stages, we have constructed eigengene network corresponding to each infection stage. We have also investigated preservation of the eigengene networks across two infection stages. The preserved eigengene networks form a higher order meta-network among the module eigengenes. Moreover, some of the meta-modules show a strong preservation during the transition of infection from one stage to another. We have also investigated the correlation between module membership (MM) values of genes with the preservation pattern of consensus modules.

## Methods

**Fig. 1:**
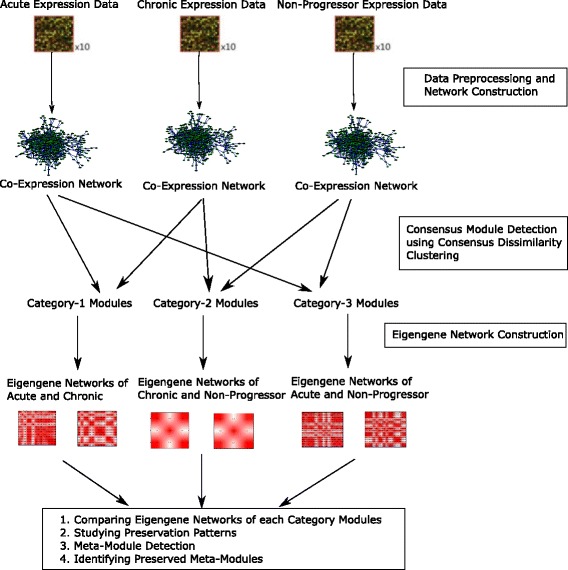
Overview of the whole framework for the present analysis

In this section, we present our proposed model to detect and analyze the modules that are shared by two or more networks (also referred to as consensus modules) across acute, chronic and non-progressor stages of HIV-1 progression. To identify consensus modules, we have utilized the popular WGCNA [24] framework. Figure 1 outlines our approach for identifying preservation affinity in consensus modules among stages of HIV-1 progression.

### Dataset used

In our present work, we have downloaded the HIV-1 microarray dataset from the Gene Expression Omnibus (GEO) database, submitted by Hyrcza MD, et al. with GEO Series accession no GSE6740 (http://www.ncbi.nlm.nih.gov/geo). It comprises of a stage-specific gene expressions of CD4+ T and CD8+ T cells from a cohort of untreated HIV-1 infected individuals and the dataset has been extracted from 10 gene chips (five gene chips from CD4+ T cells and five gene chips from CD8+ T cells, respectively) for each of the three HIV-1 stages viz. early HIV-1 infection (acute) samples, chronic infection HIV-1 samples, non-progressor HIV-1 infections samples with low or undetectable viral loads, and uninfected samples. All of the categories of datasets (acute, chronic, non-progressor, and uninfected) consist of 10 samples and 22283 genes.

### Dataset preprocessing

In addition to the expression dataset acquired from the series matrix files, we have worked out on the CEL files available in the GEO database as mentioned above. At the outset, to winnow out the outliers and for reducing the data dimensionality for computational convenience, the Affy package in the Bioconductor toolbox of R statistical software has been employed here. We extracted the expressed genes from three expression datasets corresponding to acute, chronic, and non-progressor stages that are available in the CEL files through execution of the mas5calls() function [25] of Affy package. The mas5calls() function executes Wilcoxon signed rank-based algorithm for detection and comparison calls on microarray gene expression data. Detection calls are used to find whether the transcript of a gene is present or absent. For performing detection call the intensity differences of perfectly and mismatched probes are used. Comparison call uses the differences between target genes and perfectly matched probes intensities to define the studied genes as increasing, marginally increasing, marginally decreasing, decreasing, or exhibit no expression change at all. One-sided Wilcoxon signed rank test is utilized to obtain “*p*-value” which is compared with two significance level *α*_1_ and *α*_2_. In this article, we have observed the detection call for *α*_1_ =0.05. Thus, here a gene is said to be present in a sample if the associated “*p*-value” is less than 0.05. Now, a gene is called expressed in all samples, if it is present in all of the samples. This exposes 6521, 5939, and 6939 expressed genes for the acute, chronic and non-progressor stages, respectively. The list of Genes expressed in different stages of HIV-1 infection are available in Additional file 1 and the genes exclusively expressed in different stages of HIV-1 infection are listed in Additional file 2.

**Fig. 2:**
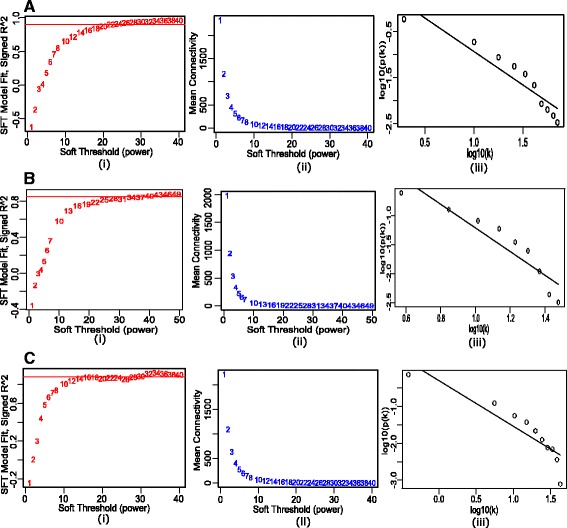
Scale Free Topology (SFT) Transformation Plots for HIV-1 infection stages. **a** acute dataset **b** chronic dataset **c** non-progressor dataset

In the next step, we transformed the expression dataset corresponding to all the stages of HIV-1 progression in a multi-set format, by uniting dataset of two stages at a time. To prepare the multi-set datasets, and to restrict our analysis to the most connected genes (i.e. genes which have high correlations in their expression profiles) and to speed up calculations when it comes to module detection, at first, we have employed the Scale-free Topology Criterion proposed by Zhang and Horvath [24]. It is observed that at soft threshold power (*β*) value of 24, the acute stage expression dataset with 6521 expressed genes satisfies scale-free topology criterion, as the scale-free topology model fitting index *R* ^2^, reaches a high threshold value (0.9), approximately (Fig. 2 a(i) and (ii)). A linear relationship between log(*p*(*k*)) and log(*k*), where *p*(*k*) is the probability of the nodes having connectivity k, further confirms that the network is transformed into a scale free network at *β* value of 24, approximately (Fig. 2 a(iii)). Applying same methodology, we have observed that the chronic stage and non-progressor stage expression datasets, approximately attained their scale-free topology criterion at *β* = 40 and *β* = 30, respectively. Next, we have calculated the connectivity of each expressed gene to all other genes for all the three expressed gene expression datasets by execution of softConnectivity() function of WGCNA package taking the (*β*) value as an argument. Thereafter, the 5600 most connected genes were extracted by computing the connectivity rank of all of the expressed genes from all the three expression datasets separately. We have also discarded the housekeeping genes which are expressed in all the samples and not associated with HIV-1 infection, from the most connected genes for our analysis. For this, first, we have selected genes that are expressed in all the samples. Next, we excluded those, which does not belong to HIV Dependency Factors (HDFs) sets (Brass et al. [26], Konig et al. [27], Zhou et al. [28]) and also do not interact with HIV-1 (from “HIV-1 Human Protein Interaction Database” (HHPID) dataset [29]) and also not included in any predicted interaction sets. For preparing predicted interaction set, we have taken union of all computationally predicted interactions from Tastan et al. [30], Dyer et al. [31], Doolittle et al. [32], Mukhopadhyay et al. [33, 34].

### Adjacency matrix and connectivity of a network

A network can be interpreted with an adjacency matrix *A* *d* *j*=[*M*_*ij*_] which indicates how nodes are connected among themselves. A gene co-expression network can be represented through a symmetric adjacency matrix consisting of *n*×*n* elements where each node in the network is a gene [35].

In an unweighted network, an element *M*_*ij*_ of the adjacency matrix gets a value 1, if nodes *i* and *j* are connected (adjacent), or 0, if the nodes are not connected. In a weighted network, 0≤*M*_*ij*_≤1 corresponds to the connection strength between the nodes *i* and *j*.

1 $$\begin{array}{*{20}l} 0 \leq M_{ij} \leq 1, \\ M_{ij} = M_{ji},\\ M_{ii}=1. \end{array} $$

Here, we have constructed gene co-expression network for all the stages of HIV-1 dataset represented in a multi-set format by computing the Spearman correlation for every pair of genes of the gene expression profile matrices.

### Transformation of the adjacency matrix

To emphasize the large adjacencies at the expense of low ones and to satisfy scale free topology criteria, we raised all the correlation values of the adjacency matrix to a fixed power *β* through power transformation law [24]

2 $$ Power_{ij}(Adj, \beta) = M^{\beta}_{ij}.  $$

The value of *β* for power transformation law is the same as soft threshold power (*β*) that we have already obtained in the “Dataset preprocessing” Section.

### Topological Overlap Matrix (TOM) based similarity measure

A major objective of network analysis is to identify groups, or modules of densely interconnected genes which can be revealed by exploring similarity patterns in connection strengths, or high “topological overlap” of among genes. The Topological Overlap Matrix (TOM) based similarity measure [36–38], which indicates how two genes are similar in terms of the commonness of genes they are connected to, has been employed in our present analysis.

TOM is expressed as

3 $$ TOM_{ij}(Adj)=\frac{\sum_{k \neq i,j}M_{ik}M_{kj}+ M_{ij}}{min\left(\sum_{k \neq i}M_{ik},\sum_{k\neq j}M_{jk}\right) +1- M_{ij}}.  $$

### TOM based dissimilarity measure

TOM based similarity matrix can be easily transformed into a dissimilarity matrix by applying the following equation:

4 $$\begin{array}{@{}rcl@{}} D_{ij}&=& Dissim_{ij}(TOM(Adj))\\ &=& 1-TOM_{ij}(Adj). \end{array} $$

### Quantile transformation

Topological Overlap Matrices (TOMs) of distinct datasets may possess different statistical features. For example, the TOM in the acute dataset may be systematically higher than the TOM in the chronic dataset. As consensus is expressed as the component-wise minimum of the two TOMs, a bias may result. Here, we illustrate a simple scaling that extenuates the effect of different statistical properties to some extent. We scale the chronic TOM such that the 95^*th*^ percentile equals the 95^*th*^ percentile of the acute TOM through Quantile transformation [21], which takes multiple TOMs of the same dimension as input and yields a single TOM whose component *Q* *u* *a* *n* *t*_*q*,*ij*_ is the *q* ^*th*^ Quantile of the corresponding components $TOM^{(1)}_{ij},TOM_{ij}^{(2)} $ of the input matrices, computed as follows:

5 $$ \begin{aligned} &Quant_{q,ij} \left(TOM^{(1)},TOM^{(2)}\right)\\ &\quad=quantile_{q} \left(TOM_{ij}^{(1)},TOM_{ij}^{(2)}\right). \end{aligned}  $$

Here, *T* *O* *M* ^(*s*)^, denotes the TOM of the dataset s.

**Fig. 3:**
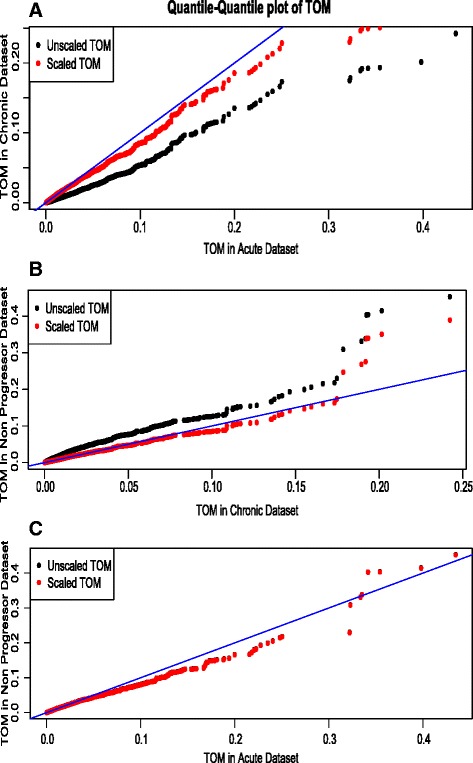
Quantile-Quantile plot of the TOMs for all pairs of datasets. **a** category–1 stages pair **b** category–2 stages pair **c** category–3 stages pair. The black points are TOMs before scaling, and the red points are TOMs after scaling. The closer the points lie to the reference line shown in blues, the closer is the distribution of the TOM values in the two data sets

To see what the scaling achieves, we form a quantile-quantile plot (Fig. 3) of all the pair of stages (e.g., acute-chronic) topological overlaps before and after scaling. From Fig. 3 a, it is clearly visible that scaling changes the chronic TOM moderately, and brings it closer to the reference line shown in blue.

#### Consensus networks

A consensus network can be constructed from the co-expression networks expressed through adjacency matrices in such a way that, two nodes are connected with each other if and only if, all of the input networks ‘agree’ on that connection. Thus, consensus network is defined as [21]:

6 $$\begin{array}{@{}rcl@{}} &&Consensus_{ij} \left(TOM^{(1)},TOM^{(2)},\ldots\right)\\ &&\quad= Min_{ij} \left(TOM^{(1)},TOM^{(2)},\ldots\right),\\ &&\text{where}, Min_{ij} \left(TOM^{(1)},TOM^{(2)},\ldots\right)\\ &&\quad= min\left(TOM_{ij}^{(1)},TOM_{ij}^{(2)},\ldots\right). \end{array} $$

#### Consensus modules

Modules in the consensus network are termed as consensus modules. In our present work, we have constructed consensus modules using pairwise gene dissimilarity measure defined analogously to Eq. (4):

7 $$  Dissim\!\left(\!\left. Consensus\!\left(\!TOM\left(Adj^{(1)}\right),TOM\left(Adj^{(2)}\right),\ldots\right)\right.\right),  $$

as input to the average linkage hierarchical clustering. The branches originating from the resulting cluster tree (Dendrogram) are referred to as consensus modules. We have utilized a dynamic tree cut algorithm [39] for this purpose. Please note that here we have used the hierarchical clustering algorithm to group genes whose expression profiles are highly correlated across samples for a pair of stages. The alternative clustering procedures can also be employed to group the genes. In this article, we have followed the procedure described in [21] to perform such grouping.

### Module summarization by Eigengene network

After constructing the consensus modules using hierarchical clustering technique as described above, we have summarized each consensus module expression profile by one representative gene: the module eigengene. Module eigengene is defined as the first right singular vector of a module expression matrix. Let, $C^{(k)} =(c_{ij}^{(k)})$ refers to the gene expression data corresponding to module *k*, where index *i*=1,2,…,*p* corresponds to the module genes and the index *j*=1,2,…,*q* corresponds to the microarray samples, and each row of *C* ^(*k*)^, has been standardized to mean 0 and variance 1. The singular value decomposition of *C* ^(*k*)^[p x q] is defined as:

8 $$ C^{(k)} = UDV^{T},  $$

where, the columns of the orthogonal matrices *U*=(*u*_1_,*u*_2_,…,*u*_(*min*(*p*,*q*))_) and *V*=(*v*_1_,*v*_2_,…,*v*_(*min*(*p*,*q*))_) are the left- and right-singular vectors, respectively, and *D*=(*d*_1_,*d*_2_,…,*d*_(*min*(*p*,*q*))_) is a diagonal matrix containing singular values. Incorporating terminology from [17, 40–42], the first column of *V* ^(*k*)^ is referred to as the Module Eigengene:

9 $$ ME^{(k)} = v_{1}^{(k)}.  $$

Let, *M* *E*_*I*_ and *M* *E*_*J*_ denote the module eigengenes of the *I* ^*th*^ and *J* ^*th*^ modules, respectively, then the connection strength between eigengenes *M* *E*_*I*_ and *M* *E*_*J*_ is expressed as:

10 $$ M_{Eigen,IJ}= \frac{1+cor(ME_{I},ME_{J})}{2}.  $$

Eigengenes of different modules of a gene co-expression network often exhibit correlations which we have used to constitute eigengene network [21]: *A* *d* *j*_*Eigen*_, which is defined as follows:

11 $$ Adj_{Eigen}= (M_{Eigen,IJ}).  $$

Eigengenes of different consensus modules often exhibit correlations which we have used to constitute consensus eigengene networks:

12 $$ Cons_{Eigen}=\left(Adj^{(1)}_{Eigen},Adj_{Eigen}^{(2)},\ldots\right).  $$

### Detecting meta-modules from Eigengene networks

After constructing the eigengene network, a module detection algorithm can be employed to detect modules in the eigengene networks that are referred to as meta-modules. The dissimilarity measure utilized here to detect such meta-modules is defined as [21]:

13 $$ Dissim_{IJ} (Adj_{Eigen}) = \frac{1-cor(ME_{I},ME_{J})}{2},  $$

where *c* *o* *r*(*M* *E*_*I*_,*M* *E*_*J*_) refers to the correlation between the module eigengenes of *I* ^*th*^ and *J* ^*th*^ modules. We have used this the dissimilarity matrix as input to the average linkage hierarchical clustering, resulting in a cluster tree of modules (represented by eigengenes) and the branches of the cluster tree are referred to as meta-modules in our application.

### Detecting consensus meta-modules

From the consensus eigengene network constructed through the method described above, a module detection algorithm can be employed again. The modules in the consensus eigengene networks hence detected are referred to as consensus meta-modules. The dissimilarity measure utilized here to detect such meta-modules is analogous to Eq. (13) and expressed as:

14 $$ Dissim (Cons\left(Adj_{Eigen}^{(1)},Adj_{Eigen}^{(2)},\ldots\right).  $$

The branches emanating from the cluster tree of modules resulting from average linkage hierarchical clustering using the above dissimilarity matrix as input correspond to consensus meta-modules in our application.

### Identifying overlaps among the consensus modules

In the present article, we have also computed the overlaps among the identified consensus modules by taking two categories of modules at a time. To measure the overlap we have used Jaccard-based similarity metric defined as follows:

15 $$ O_{i,j} = \frac{| M_{i} \cap M'_{j} |}{| M_{i} \cup M'_{j} |},  $$

where *M*_*i*_ ∈ category-*i* module, while $M^{\prime }_{j} \in $ category-*j* module. For each pair of modules we have computed the overlap and constructed an overlap matrix as *O* *v* *e* *r* *l* *a* *p*_*mat*_=[ *O*_*i*,*j*_]_*m*×*n*_. The overlap scores for category-*i* module *M*_*i*_ and category-*j* module $M^{\prime }_{j}$ are defined as

16 $$\begin{array}{@{}rcl@{}} OvScore_{M_{i}}&=&max^{n}_{j=1}Overlap_{M_{i},M'j}, and \\OvScore_{M^{\prime}_{j}}&=&max^{m}_{j=1}Overlap_{M_{i},M'j}, \end{array} $$

where *n* and *m* are the numbers of category-*i* and category-*j* modules, respectively. The OvScore metric of a module indicates the proportion of involvement of it in two other categories of modules.

### Identifying preservation pattern in consensus modules among HIV-1 stages

To discover the changes in preservation patterns across each category of consensus modules, we have compared the two eigengene networks, each corresponds to an HIV-1 stage in a specific category. For example, we have compiled the eigengene networks corresponding to acute and chronic stages in category-1 module.

To compare eigengene networks (Eq. (11)), we have used the measures introduced in [21]. Let $Adj^{(p)}_{Eigen}$ and $Adj^{(q)}_{Eigen}$ denote the adjacency matrices of consensus eigengene networks of stages *p* and *q*. We construct a preservation network between these two consensus eigengene networks as follows:

17 $$ Pres^{(p,q)} = Pres\left(Adj^{(p)}_{Eigen},Adj^{(q)}_{Eigen}\right),  $$

where the entries of the preservation network *P* *r* *e* *s* ^(*p*,*q*)^ are defined as:

18 $$  Pres^{(p,q)}_{I,J}\,=\,1 - \frac {\left|cor\left(ME^{(p)}_{I},ME^{(p)}_{J}\right) - cor\left(ME^{(q)}_{I},ME^{(q)}_{J}\right)\right|}{2}.  $$

Here, $ME_{I}^{(k)}$ signify the eigengene of the *I* ^*th*^ consensus module in dataset *k*. Larger values of $Pres_{I,J}^{(p,q)}$ signify more preservation of correlation pattern among module eigengenes *M* *E*_*I*_ and *M* *E*_*J*_ across two networks.

Furthermore, to investigate the preservation between module eigengenes across two networks we have computed the Scaled Connectivity *C*_*I*_(*P* *r* *e* *s* ^(*p*,*q*)^) [21] of a module eigengene $ME_{I}^{(k)}$ which is given as:

19 $$ \begin{aligned} C_{I} (Pres^{(p,q)}) = 1 - \frac {\sum_{J \ne I} \left|cor\left(ME^{(p)}_{I},ME^{(p)}_{J}\right) - cor\left(ME^{(q)}_{I},ME^{(q)}_{J}\right)\right|}{2(N-1)}. \end{aligned}  $$

*C*_*I*_(*P* *r* *e* *s* ^(*p*,*q*)^) is close to 1 if the *I* ^*th*^ module eigengene has a strong preservation pattern with the most of the other eigengenes. The density (D) [21] of the preservation network *P* *r* *e* *s* ^(*p*,*q*)^ is given by:

20 $$ \begin{aligned} D(Pres^{(p,q)}) \,=\, 1\! -\! \frac {\sum_{I} \sum_{J \ne I} \left|cor\left(ME^{(p)}_{I},ME^{(p)}_{J}\right) - cor\left(ME^{(q)}_{I},ME^{(q)}_{J}\right)\right|}{2N(N-1)}. \end{aligned}  $$

Larger values of *D*(*P* *r* *e* *s* ^(*p*,*q*)^) indicate a strong preservation of correlation patterns among the most of the eigengenes across the two networks.

### Computing module membership of genes within consensus modules

Module membership (MM) of a gene is defined as the Pearson correlation value between the expression level of a gene on the microarray and the module eigengene. The measure describes the extent of similarity between the expression level of the gene and the overall expression pattern of the module. Here, we compared the MM values of all the genes within a shared module between a pair of infection stages. In particular, we have computed the module membership values of all the genes within a shared module $M_{i}^{p_{1}-p_{2}}$, for stage *p*_1_ as follows:

21 $$\begin{array}{@{}rcl@{}} MM\_M_{i}^{p_{1}}&=& [\!v_{1},v_{2}, \ldots v_{n}], \\ \text{where},v_{j}&=& corr(ME_{i},g_{j}). \end{array} $$

Here, *M* *E*_*i*_ is the module eigengene of module *M*_*i*_, *g*_*j*_ is the expression profile of *j* ^*th*^ gene in the module, and *c* *o* *r* *r*(.) denotes the Pearson correlation operator. Similarly, we have computed the MM values of module $M_{i}^{p1-p2}$ for stage *p*2. Therefore, for a shared module between a pair of stages, we obtained two sets of MM values, each of which corresponds to one stage. Next, we merged these two sets into one set of MM values and compared this set between the preserved shared modules.

## Results and discussion

Here we report the results of our eigengene based analysis of consensus modules identified at different stages of HIV-1 progression. For the rest of the paper, we will use the term category-1 modules for consensus modules of acute and chronic stages, category-2 modules for consensus modules of chronic and non-progressor stages and category-3 modules for consensus modules of acute and non-progressor stages.

### Overlaps among the expressed genes

**Fig. 4:**
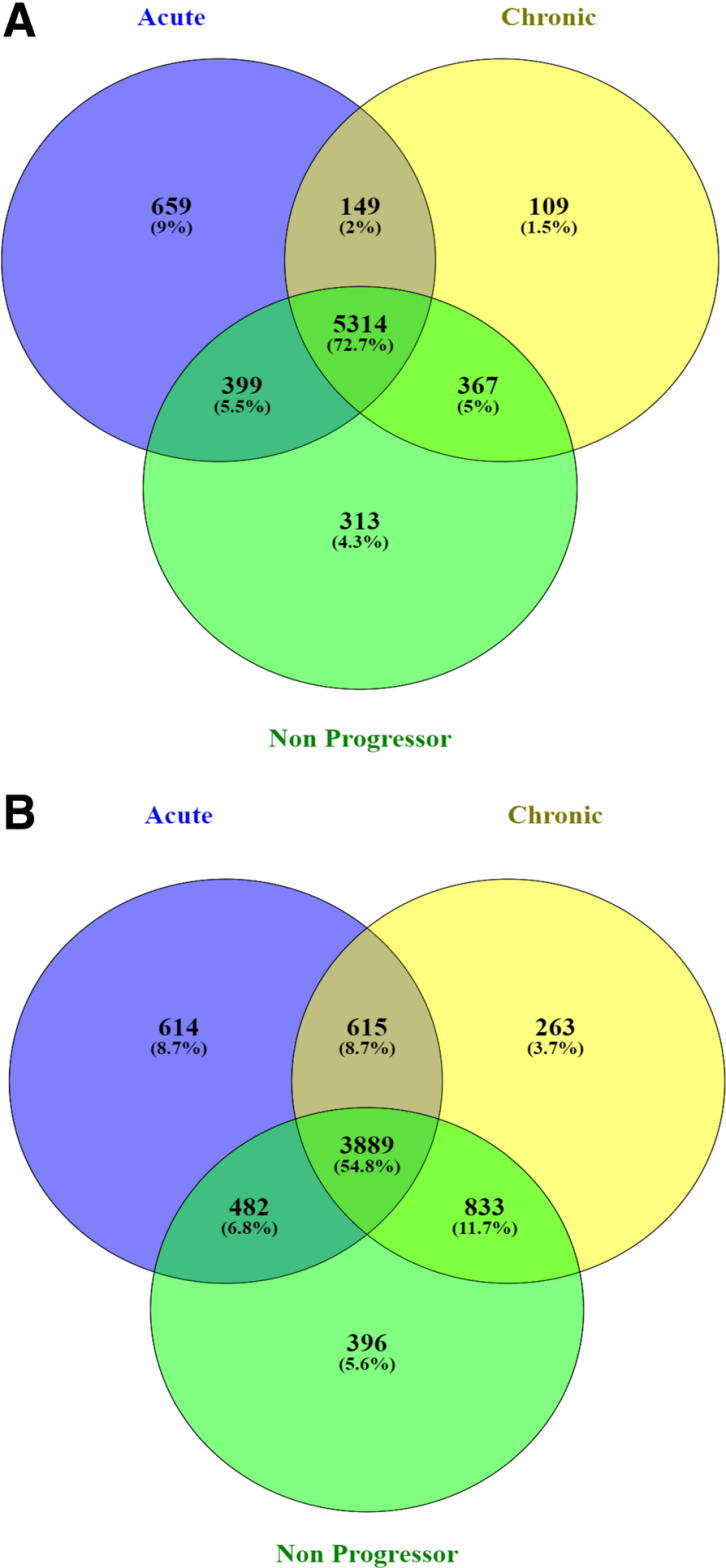
**a** Venn Diagram showing the count of the expressed genes among HIV-1 stages. We have observed 6521, 5939, and 6939 expressed genes for the acute, chronic and non Progressor stages, respectively. **b** Venn Diagram showing the count of 5600 Most Connected Expressed Genes (MCEGs) observed in all of the HIV-1 Stages

We have observed the overlaps among the selected expressed genes in acute, chronic and non-progressor stage. Figure 4 shows the overlaps among 6521 selected genes of acute stage, 5939 selected genes of chronic stage and 6393 selected genes of non-progressor stage. It can be noticed from Fig. 4 a that all the stages share a good amount of common genes (72.7%) among themselves. A relatively small number of expressed genes (109/1.5%) of chronic stage have no overlaps with the expressed genes of other stages. For consensus module detection, we have transformed the expression profiles of these expressed genes of all the stages into a multi-set format. We have chosen the 5600 most connected genes for all the stages of HIV-1 progression which we have discussed earlier in the “Methods” section. A closer look at the Fig. 4 b reveals that the number of common genes (54.8%) among the stages has been decreased from our earlier observation. The possible reasons behind this, is that the connectivities among a significant number of expressed common genes with other genes are low compared to the connectivities among expressed non-common genes with other genes. As a supplementary information to the interested readers, we have included Additional files 3 and 4 which show the Venn diagrams of the expressed genes and the 5600 most connected expressed genes (MCEG), respectively, among the uninfected and three stages of HIV-1 infection.

### Identification of consensus modules

**Fig. 5:**
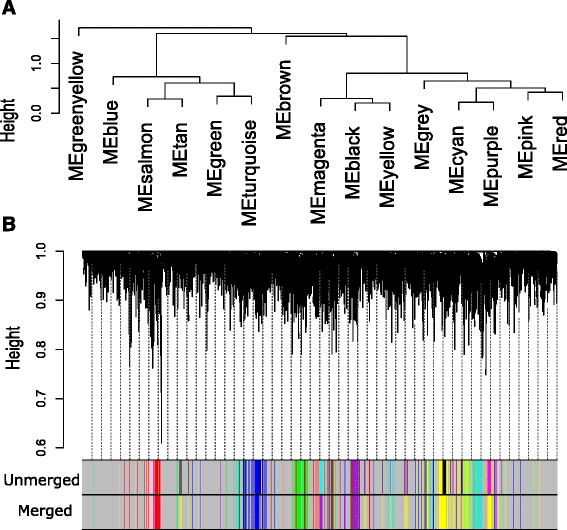
**a** Hierarchical clustering tree for category-1 consensus modules. Each consensus module is described here as a module eigengene of it (e.g. module ‘*blue*’ is represented as ‘MEblue’). **b** The dendrogram for category-1 consensus modules. Here, ‘unmerged’ color codes signify the consensus modules whereas merged denote the consensus meta-modules. In this case, three consensus modules ‘*yellow*’, ‘*black*’ and ‘*magenta*’ are grouped in ‘*yellow*’ meta-module and ‘*cyan*’ and ‘*purple*’ are grouped into ‘*purple*’ meta-module

**Fig. 6:**
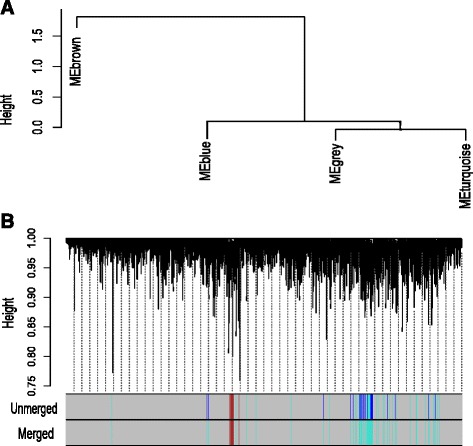
**a** Hierarchical clustering tree for category-2 consensus modules. Each consensus module is described here as a module eigengene of it (e.g. module ‘*blue*’ is represented as ‘MEblue’). **b** The dendrogram for category-2 consensus modules. Here, ‘unmerged’ color codes signify the consensus modules whereas merged denote the consensus meta-modules. Here, consensus modules ‘*blue*’ and ‘*turquoise*’ are grouped into meta-module ‘*turquoise*’

**Fig. 7:**
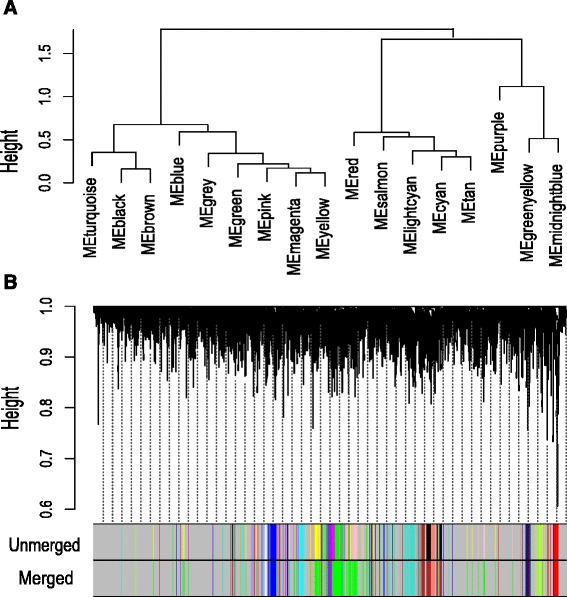
**a** Hierarchical clustering tree for category-3 consensus modules. Each consensus module is described here as a module eigengene of it. (e.g. module ‘*blue*’ is represented as ‘MEblue’). **b** The dendrogram for category-3 consensus modules. Here, ‘unmerged’ color codes signify the consensus modules whereas merged denote the consensus meta-modules. Here, consensus modules ‘*black*’ and ‘*brown*’ are grouped into meta-module ‘*brown*’, consensus modules ‘*green*’, ‘*pink*’, ‘*magenta*’ and ‘*yellow*’ are grouped to meta-module ‘green’

We have utilized the consensus dissimilarity measure Eq. (7) in average linkage hierarchical clustering method to detect consensus modules. We take a pair of stages at a time and identify consensus modules from the expressed genes. The identified modules are given the same type of color code. The genes which are not assigned to any of the modules are labeled as gray color. We have obtained 14 consensus modules of category-1, as shown in Fig. 5. Similarly, we have obtained 3 category-2 and 16 category-3 modules (shown in Figs. 6 and 7). We have summarized each category modules by their corresponding module eigengenes through Eq. (10) and constructed an eigengene network among them using Eq. (11). For each category of consensus modules there are two sets of genes each corresponding to a specific HIV-1 infection stage. We have included the list of genes which are involved in the formation of all categories of consensus modules in Additional files 5, 6, 7, 8, 9 and 10.

It is worth noting that the number of category-2 modules for the chronic and non-progressor stages pair is relatively small and major genes didn’t participate in modules formation (as they fall in gray module). This indicates that the commonness between the expression patterns of chronic and non-progressor is much lower than the other pair of stages.

### Overlaps among the consensus modules

To detect the overlaps among the identified consensus modules, we have applied Eqs. 15 to 16 and obtained the overlap scores (OvScore) for all categories of consensus modules.

**Fig. 8:**
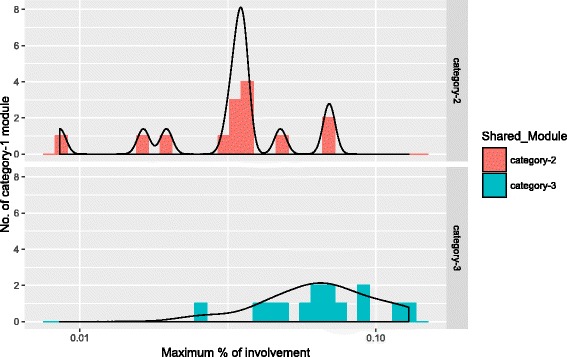
Figure shows percentage of involvement of category–1 modules in category–2 and category–3

**Fig. 9:**
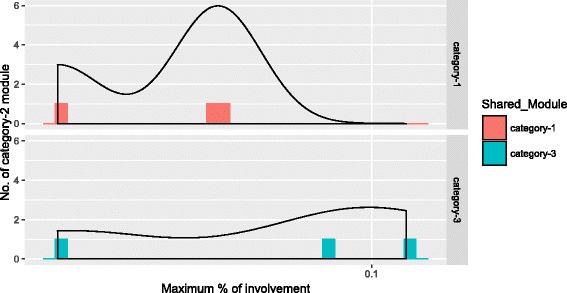
Figure shows percentage of involvement of category–2 modules in category–1 and category–2

**Fig. 10:**
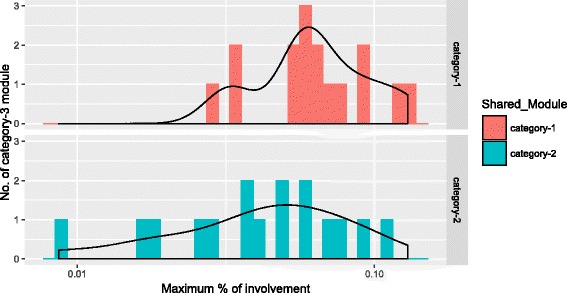
Figure shows percentage of involvement of category–3 modules in category–1 and category–2

**Fig. 11:**
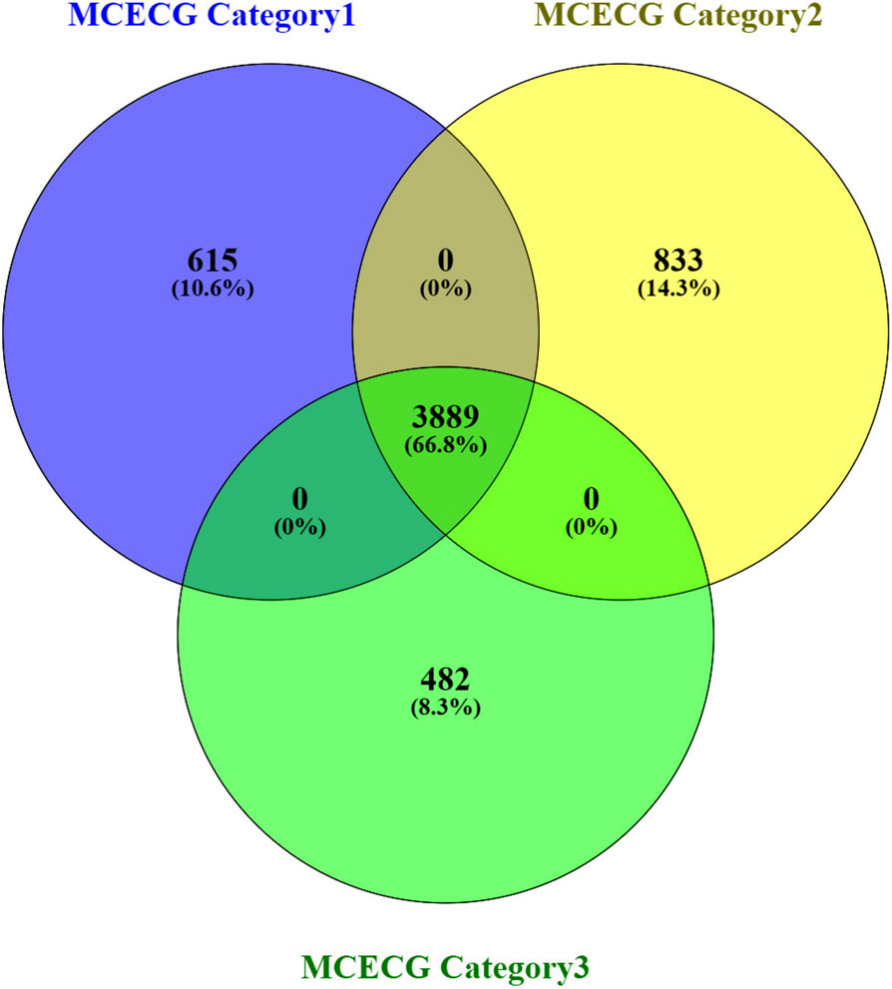
Venn diagram showing the count of the Most Connected Expressed Common Genes (MCECG) among acute-chronic (i.e. category–1), chronic–non progressor (i.e. category–2) stages and acute–non progressor (i.e. category–3) stages

Figures 8, 9 and 10 show the distribution of three categories of modules with their respective OvScore values. It is noticed from these figures that there is very little involvement among the three categories of modules. Most of the modules in each category have low OvScore. To investigate, whether these results have any correlation with the number of common genes that are involved in the consensus modules construction in each pair of the stages, we have performed the following analysis. We have drawn a Venn diagram in Fig. 11 to show the overlap among the common genes which are involved in the consensus modules construction for each pair of stages. It is observed from the figure that 66.8% genes are common among them. The complete list of all overlapped genes for Fig. 11 is provided in Additional file 11. The genes which are preserved between the stages across all the consensus modules are also listed in Additional file 12. After removing the housekeeping genes from the most connected expressed genes for each stage, we have found 57.05% genes are common among the genes involved in consensus module construction. Among those common genes we have also searched for the Immune Regulatory genes [43] and found some of them between the stages across all the consensus modules. We have collected and compiled a list of immune regulatory genes from Immunology Database and Analysis Portal (ImmPort), Immunogenetic Related Information Source (IRIS) and Immunome Database available in InnateDB [44]. The list of such Immune Regulatory genes preserved among the HIV-1 stages across all the consensus modules is available in Additional file 13 and the exclusive set of Immune Regulatory genes expressed in different stages of HIV–1 infection are listed in Additional file 14.

**Fig. 12:**
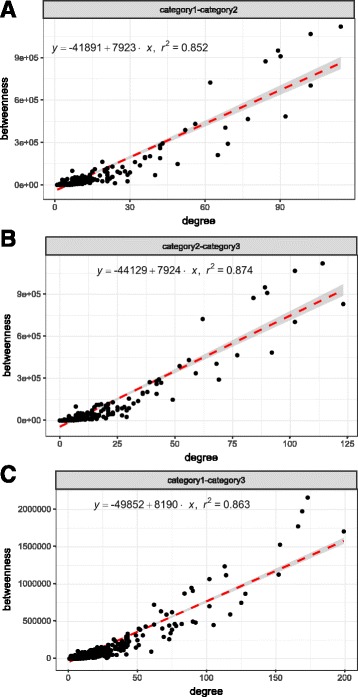
Scatter plots of degree vs. betweenness centrality of the shared genes among all categories of modules. **a** category-1 – category-2 **b** category-2 – category-3 **c** category-1 – category-3

Furthermore, to explore the characteristics of the shared genes belonging to the consensus modules of each pair of stages, we have performed the following analysis. We have investigated the degree and betweenness centrality of the genes considering the whole human genome as an interaction network. Figure 12 (a), (b) and (c) show the scatter plots of degree vs. betweenness centrality of these shared genes. It can be observed from the figure that, there exists a strong correlation between degree and betweenness centrality of the genes in each category. For shared genes between category-1 and category-3, *R* ^2^ value (0.883) is slightly more than the shared genes of other pair of categories (for category-2 and category-3: 0.874, for category-1 and category-2: 0.852). Some shared genes emerge as both hub (high degree) and bottleneck (high betweenness centrality). For example, genes: ‘ACTB’, ‘EEF1E1’, ‘CALM1’, ‘HSP90AA1’, ‘RAC1’, ‘STAT1’, ‘CSNK2A1’ and ‘STAT3’ have degrees 102, 89, 114, 90, 92, 77, 152 and 102 and betweenness centrality 1.066E+06, 9.49E+05, 9.9E+05, 1.118E+07, 4.85E+06, 4.65E+06, 1.12E+06, and 7.022E+06, respectively. To further explore the biological relevance of the shared genes, we have searched Gene Ontology (GO) terms and KEGG pathways that are associated with those genes. Table 1 summarizes the results for the shared genes of each category modules.

**Table 1:** Gene Ontology (GO) term and KEGG pathway of the shared genes of each category modules

Category pair	GO-Term (GO ID) (*p*-Value)	KEGG Pathway (*p*-Value)
Category-1–category-2	Translational initiation (GO:0006413) (*p*-value: 7.5E-19)	Ribosome (3.1E-7)
Category-1–category-3	Viral process (GO:0016032) (*p*-value: 8.0E-33)	HTLV-I infection (8.8E-12)
Category-2–category-3	Viral transcription(GO:0019083)(*p*-value: 2.4E-14)	Phagosome (4.7E-5)

### Preservation of consensus modules between each pair of stages

For each pair of stages, consensus modules are identified by using a consensus dissimilarity measure (Eq. (14)) which is utilized in the hierarchical clustering algorithm. The eigengene networks among the consensus modules represent how the characteristic expression patterns of modules are correlated with each other in a particular stage. We have constructed eigengene network corresponding to each infection stage for each category of consensus modules. For example, in category-1 module we have compiled the eigengene networks corresponding to acute and chronic stages. We have employed Eqs. 17 to 18 for comparing these two eigengene networks to know the changes in preservation patterns across each category of consensus modules.

**Fig. 13:**
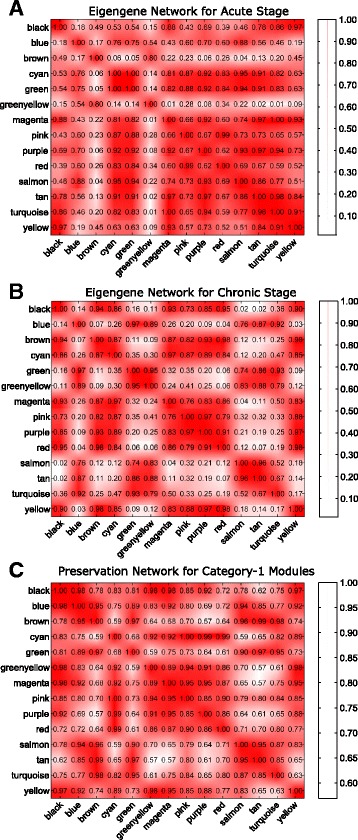
Figure shows the heatmaps of eigengene networks for 14 category–1 modules. Panel **a** shows the eigengene network for acute stage and Panel **b** shows the same for chronic stage. Panel **c** shows the preservation network for the category-1 modules

**Fig. 14:**
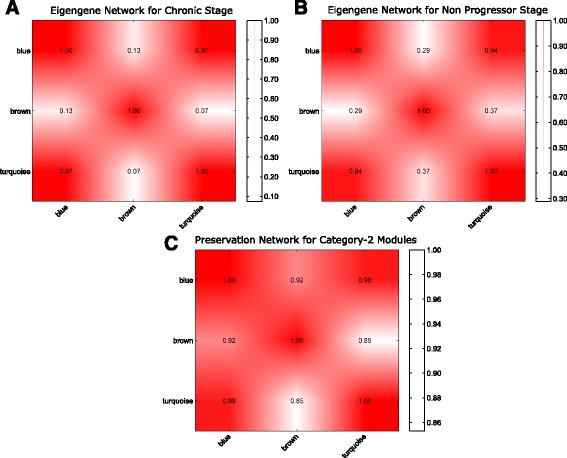
Figure shows the heatmaps of eigengene networks for three category-2 modules. Panel **a** shows the eigengene network for chronic stage and Panel **b** shows the same for non progressor stage. Panel **c** shows the preservation network for the category-2 modules

**Fig. 15:**
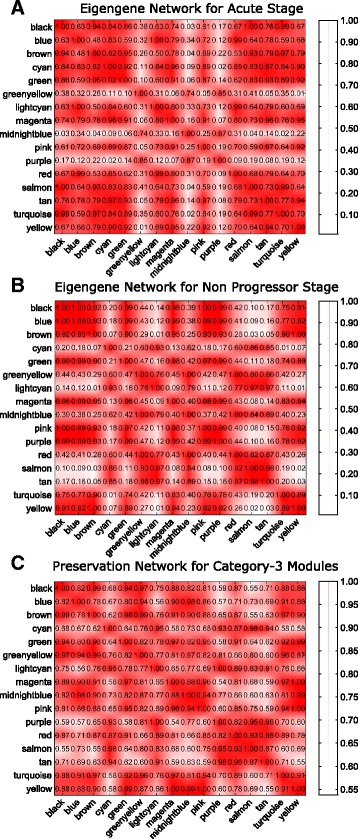
Figure shows the heatmaps of eigengene networks for 16 category-3 modules. Panel **a** shows the eigengene network for acute stage and Panel **b** shows the same for non progressor stage. Panel **c** shows the preservation network for the category-3 modules

Figure 13(a) and (b) show the heatmap of eigengene networks of category-1 modules corresponding to acute and chronic stages. Figure 13(c) shows the preservation network for the same. It can be noticed from this Figure that five consensus modules ‘greenyellow’, ‘magenta’, ‘purple’, ‘pink’, and ‘red’ retain their pairwise correlation pattern across acute and chronic stages. In other words, these modules preserve their expression patterns across acute and chronic stages. For category-2 and category-3 modules the heatmaps of eigengene networks and preservation networks are shown in Figs. 14 and 15, respectively. From Fig. 15(c) we noticed that there are several clusters of modules exist that preserve their expression pattern across acute and non-progressor stages. For example, purple, red, yellow and tan modules retain their pairwise correlation pattern same across acute and non-progressor stages. Another example includes black, blue and brown modules, or magenta, midnightblue and pink modules. For category-2 modules blue and brown modules have a same correlation pattern across chronic and non-progressor stages.

Additionally, for investigating the preservation between module eigengenes across two networks we have computed the Scaled Connectivity (Eq. (19)) of the module eigengenes for each category of modules and the density (Eq. (20)) of their preservation network.

**Fig. 16:**
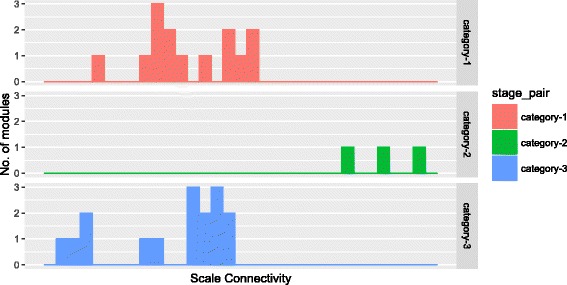
Figure shows the distribution of scale connectivity values of consensus modules for three categories

Here, we report the results of preservation measures which are applied to the three categories of modules. Figure 16 shows the distribution of each category of modules with scale connectivity (C) values. As can be seen from the figure, category-1 and category-3 modules show similar types of distribution over the values of *C*. The density (D) value for category-1 module is 0.7956 whereas for category-3 module the value is 0.8060. The value *D* for category-2 modules is much higher (0.9195) than that for category-1 and category-3. The possible reason may be that the number of shared modules for chronic and non-progressor stages is only three.

To assess the significance of preservation among the shared modules, we have performed the following statistical test. For this, we have constructed three categories of shared modules randomly from the identified expressed genes of three infection stages. Thus, we obtained 14 random modules for category-1, 3 for category-2 and 16 for category-3. Random modules of each category are constructed by selecting genes randomly from the common expressed genes of a pair of stages. To investigate the preservation pattern of the constructed random modules, we computed the eigengene network and preservation matrix for each stage. From this, scale connectivity (C) values are computed for each category of random modules. We compared the C values of random modules with the original modules using Wilcoxon Ranksum test. The resulting *p*-values (7.4678e-06 for category-1 modules, 1.1296e-05 for categoty-3 and 3.2487e-05 for categoty-2) are very low, which signify the preservation of expression pattern between each pair of infection stages is statistically significant.

This suggests that there exists a strong preservation in the overall expression pattern of each category of modules.

### Higher order organization of consensus modules

**Fig. 17:**
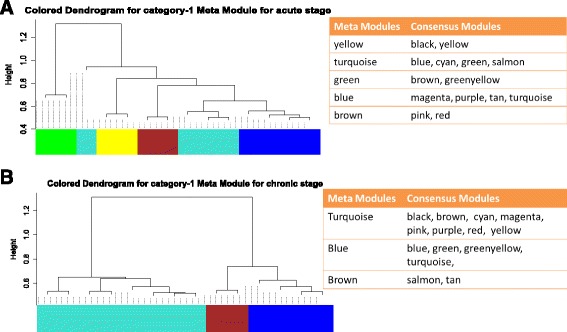
Figure shows higher order organization of category-1 modules in acute and chronic stages. Hierarchical clustering tree of consensus modules according to acute stage is shown in panel **a**. Panel **b** shows the same for chronic stage. In each panel the grouping of consensus modules into meta-modules are shown

**Fig. 18:**
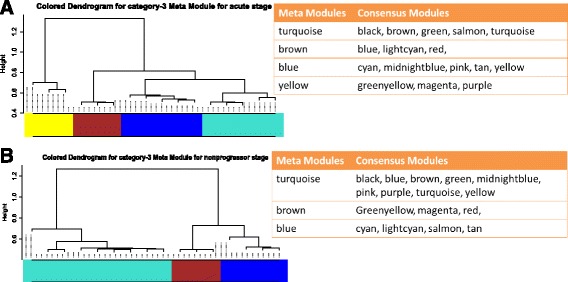
Figure shows higher order organization of category-3 modules in acute and non-progressor stages. Hierarchical clustering tree of consensus modules according to acute stage is shown in Panel **a**. Panel **b** shows the same for non progressor stage. In each panel the grouping of consensus modules into meta-modules are shown

From Figs. 13, 14 and 15, it can be noticed that the shared modules not only preserve their correlation patterns across a pair of infection stages but also form groups or clusters corresponding to each infection stage. For example, in category-1 modules, magenta, green, purple and red modules have high correlation score among them in chronic stage. Similarly, salmon, tan and turquoise modules show high correlation among them in acute stage. This suggests to form a higher order structure of modules that reflects the relationship among them. To investigate the relationship among the modules in each stage, we have performed hierarchical clustering on each category of modules, by using the dissimilarity measure shown in Eq. (13). The identified meta-modules in each category, signify the association among the consensus modules. Figures 17 and 18 show the hierarchical clustering tree to detect meta-modules for category-1 and category-3 modules, respectively. For consensus modules of category-2, no meta-modules are found in both chronic and non-progressor stages. For category-1, we observed five meta-modules (yellow, turquoise, green, blue, and brown) in acute stage and three meta-modules (turquoise, blue, brown) in chronic stage. In category-3, four meta-modules (turquoise, brown, blue, and yellow) are identified in acute stage, while three meta-modules (turquoise, brown, and blue) are found in non-progressor stage. Such groupings of consensus modules at each stage represent a strong correlation of expression patterns among the modules. From Fig. 17(a) and (b), one can observe the preservation of meta-modules across two stages. For example, in category-1, the first (yellow) and fifth (brown) meta-modules of acute stage are fully preserved in chronic stage. The second meta-module (turquoise) of acute stage is partially preserved in chronic stage. Similarly, from Fig. 18, it can be seen that the first (turquoise) and fourth (yellow) meta-modules of acute stage are highly preserved in non-progressor stage.

From Fig. 13(c), we noticed that a good amount of preservation exists among the consensus modules. It is also observed from Figs. 17 and 18, that the preservation exists in stage-specific meta-modules. So, it is tempting to investigate the preservation pattern among the consensus meta-modules. We detect consensus meta-modules by following the same methodology for consensus module detection. Identified eigengene networks for two stages are clustered using hierarchical clustering by using the dissimilarity measure mentioned in Eq. (14) to form consensus meta-modules. We have found 11 consensus meta-modules for category-1, 2 consensus meta-modules for category-2 and 12 consensus meta-modules for category-3. Figure 5 shows the hierarchical clustering tree for consensus meta-module detection. We noticed in the figure that modules black, yellow and magenta are merged to form a one consensus meta-module whereas cyan and purple form another consensus meta-module. Similarly, Figs. 6 and 7, show the consensus meta-module formation for category-2 and category-3 modules, respectively. Such type of meta-modules represents a grouping of consensus modules between two stages. The difference between the consensus meta-module and simple meta-module is that the consensus meta-modules are constructed from consensus modules by considering a pair of stages. It represents shared meta-modules across two infection stages.

### Consistency of module membership with the preservation pattern

**Fig. 19:**
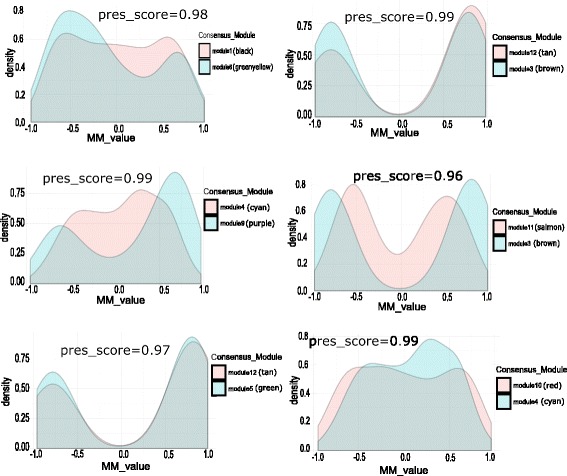
Figure shows density plots of module membership (MM) values of genes in preserved modules of category-1. Each plot describes the distribution of MM values of genes within a pair of preserved modules. Here, the density plots are shown for six pair of preserved modules (preservation score ≥ 0.95)

**Fig. 20:**
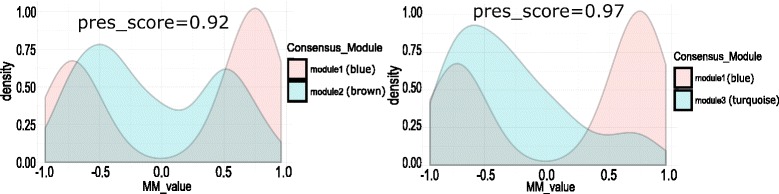
Figure shows density plots of module membership (MM) values of genes in preserved modules of category-2. Each plot describes the distribution of MM values of genes within a pair of preserved modules. Here, the density plots are shown for two pair of preserved modules (preservation score ≥ 0.91)

**Fig. 21:**
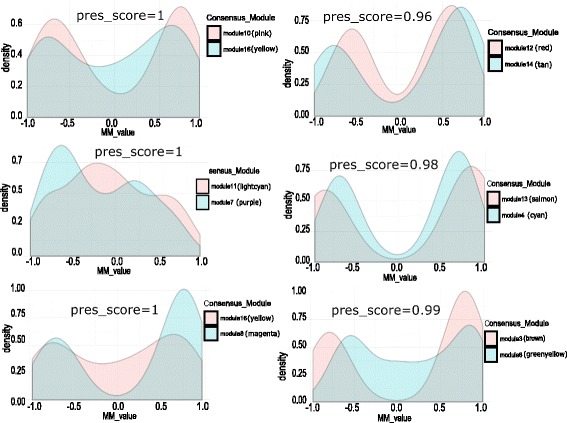
Figure shows density plots of module membership (MM) values of genes in preserved modules of category-3. Each plot describes the distribution of MM values of genes within a pair of preserved modules. Here, the density plots are shown for six pair of preserved modules (preservation score ≥ 0.95)

In this article, we have also computed the module membership (MM) values of all the genes within a consensus module for the pair of infection stages in each category of modules using Eq. (21) and compared their MM values. Figure 19 shows the comparison of MM values for each pair of preserved category-1 modules. Each panel of Fig. 19 shows a density plot of MM values for preserved category-1 models. Here, we show distribution of MM values for six pairs of category-1 modules with high preservation score. It is evident from the figure that the MM values are consistent with the preservation score of the modules. For example, two preserved shared modules (score=0.99) module 4 (cyan) and module 9 (purple) show similar patterns in the distribution of their MM values. We can observe the same consistency in category-2 and category-3 modules. Figures 20 and 21 show the comparison of MM values between preserved category-2 and category-3 modules, respectively. This suggests that module membership is consistent with the preservation pattern of consensus modules.

### Expression analysis of HIV infected individuals before and after ART

**Fig. 22:**
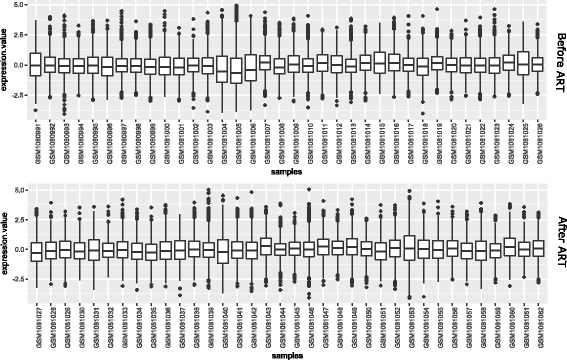
Figure shows box plots of HIV infected 36 samples before and after Antiretroviral Therapy (ART)

**Fig. 23:**
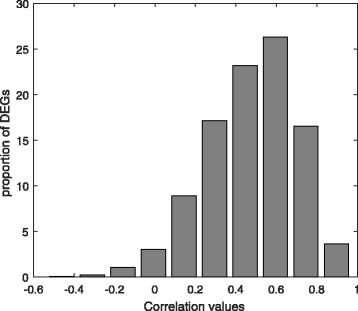
Bar plot describing proportion of DEGs with correlation values in all treated and untreated samples

An effective suppression of viral replication (≤50 copies/mL) following an increase in CD4+ T-cell counts can be observed through Antiretroviral therapy (ART) in HIV infected individuals. In this experiment, we have performed a gene expression analysis with the microarray expression dataset (GSE44228 [45]) which provides gene expression values of 36 HIV infected individuals before and after antiretroviral therapy (ART). In this analysis, we have utilized 4,157 differentially expressed genes (DEGs) identified through multivariate permutation tests, provided in [45]. We have investigated expression values the DEGs in treated and untreated samples, individually. Figure 22 shows box-plots of all the treated and untreated 36 samples. Moreover, to know which genes preserved their expression patterns in both treated and untreated samples, we have computed the Pearson correlations between the expression profiles of all the DEGs in both the samples. In Fig. 23, we have shown a bar plot which describes proportions of DEGs with correlation values. It can be observed from this figure that approximately 50% of the DEGs has correlations between 0.4 to 0.6. A small percentage (∼ 5%) of the DEGs has correlations greater than 0.8 and a very few of the DEGs exhibit negative correlations.

**Fig. 24:**
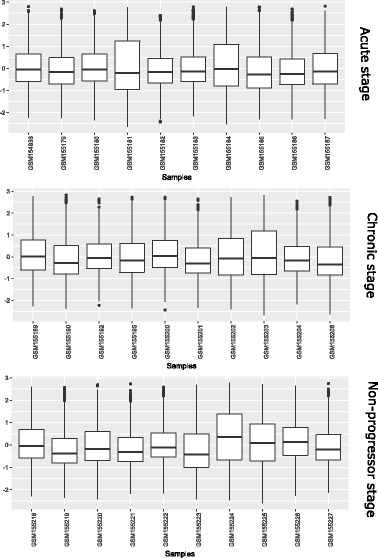
Box plots of DEGs in Acute, Chronic and Non Progressor Samples

We have also compared expression values of the DEGs in three HIV infections stages: acute, chronic and non-progressor. For this, we have collected expression profiles of the DEGs in acute, chronic and non-progressor samples. Figure 24 shows the box-plots of these samples in acute, chronic and non-progressor stages.

## Conclusions

In the present article, we have carried out a comprehensive analysis to investigate the preservation pattern of coexpression network compiled from microarray gene expression data of HIV-1 progression stages. Here, three different categories of consensus modules are identified by considering each pair of infection stages at a time. For each category, we have compiled two eigengene networks of a consensus module, corresponding to each infection stage. We have found that eigengene networks are preserved in each pair of infection stages. The preservation pattern is more prominent in category-3 (consensus modules of acute and non-progressor pair) modules. However, there exists little involvement among the consensus modules between three categories. Moreover, the number of consensus modules of category-2 is only three, which indicates the preservation of network properties between chronic and non-progressor stage is not good. However, the preservation scores of blue, brown and turquoise modules in category-2 are high, which signifies the correlation between eigengenes of each pair of modules remain the same in chronic and non-progressor stages. So, the preservation of eigengene in category-2 is high despite having low preservation of network properties between chronic and non-progressor stages.

Observing the preservation pattern of each category of modules in an individual infection stage, we have clustered the modules into groups of meta-modules. Each meta-module is identified in individual infection stage by performing hierarchical clustering which utilizes the dissimilarity measure defined in Eq. (13). The meta-modules are fully or partially preserved across a pair of infection stages. Some meta-modules in category-1 such as ‘green’ and ‘blue’ are not preserved between acute and chronic stages. List of the genes involved in those two meta modules are listed in Additional file 15. Similarly, for category-3, meta-module ‘brown’ is not preserved between acute and non-progressor stages. For category-2, no meta-modules are found and the possible reason behind this is the small number of identified consensus modules. Moreover, the preservation among the consensus meta-modules are also discovered by identifying them using the Eq. (14) from consensus modules.

Apart from the eigengene networks, the preservation among the consensus modules is also observed while comparing the module membership (MM) values of genes within the modules. In other words, the MM values are found to be consistent with the preservation pattern of eigengene networks. In most of the cases, the distribution of MM values between two preserved modules in each category shows a strong correlation. This suggests that the way in which the genes within a pair of preserved module conforms to its characteristic expression pattern is similar.

Some issues still require to be explored further. It is worth mentioning that a clear investigation of the preserved modules through biological experiments can facilitate the understanding of key players or biomarkers that are essential for HIV infection. Apart from that, different machine learning approaches like support vector machines, rule-based systems, random forests, artificial neural networks, etc. may be useful tools for capturing the preservation structure among consensus modules of different stages of HIV-1 infection. Multilabel classifications would be an important tool to predict drug resistance in HIV-1 antiretroviral therapy [46, 47]. Beside, detecting preservation patterns module-wise, it is also interesting to identify the differentially co-expressed modules across a pair of stages in HIV-1 progression. We are now working in this direction.

## Additional files

Additional file 1 List of genes expressed in different stages of HIV–1 infection. (XLSX 95.7 kb)

Additional file 2 List of genes exclusively expressed in different stages of HIV–1 infection. (XLSX 21.1 kb)

Additional file 3 Venn diagram showing the count of the expressed Genes among the uninfected and three stages of HIV–1 infection. (EPS 21.1 kb)

Additional file 4 Venn Diagram showing the count of the 5600 most connected expressed genes among the uninfected and three stages of HIV–1 infection. (EPS 210 kb)

Additional file 5 List of acute stage genes involved in category-1 (acute–chronic stage Pair) consensus module formation. (XLSX 23.0 kb)

Additional file 6 List of chronic stage genes involved in category-1 (acute–chronic stage Pair) consensus module formation. (XLSX 22.0 kb)

Additional file 7 List of chronic stage genes involved in category-2 (chronic–non-progressor stage pair) consensus module formation. (XLSX 12.0 kb)

Additional file 8 List of non-progressor stage genes involved in category 2 (chronic–non-progressor stage pair) consensus module formation. (XLSX 11.9 kb)

Additional file 9 List of acute stage genes involved in category-3 (acute–non-progressor stage pair) consensus module formation. (XLSX 21.5 kb)

Additional file 10 List of non-progressor stage genes involved in category-3 (acute–non-progressor stage pair) consensus module formation. (XLSX 21.5 kb)

Additional file 11 List of the most connected expressed common genes (MCECG) in each category of modules. (XLSX 76.2 kb)

Additional file 12 List of genes preserved in consensus modules of each category. (XLSX 11.5 kb)

Additional file 13 List of immune regulatory genes preserved in consensus modules in each category. (XLSX 9.80 kb)

Additional file 14 List of exclusive immune regulatory genes expressed in stages of HIV–1 infection. (XLSX 12.1 kb)

Additional file 15 List of the genes involved in the non–preserved category–1 meta-modules: “green” and “blue”. (XLSX 22.7 kb)

## Abbreviations

AIDS: Acquired immunodeficiency syndrome
ART: Antiretroviral therapy
Category–1 Modules: Consensus modules of acute and chronic stages
Category–2 Modules: Consensus modules of chronic and non-progressor stages
Category–3 Modules: Consensus modules of acute and non-progressor stages
DEG: Differentially expressed genes
GEO: Gene expression omnibus
GO: Gene ontology
HIV: Human immunodeficiency virus
HDFs: HIV dependency factors
HPID: HIV-1 human protein interaction database
MM: Module membership
ME: Module Eigengene
MCEG: Most connected expressed genes
MCECG: Most connected expressed common genes
OvScore: Overlap score
TOM: Topological Overlap Matrix
WGCNA: Weighted gene co-expression network analysis
SFT: Scale-Free Topology
